# Biogeography of the fish pathogen *Aeromonas salmonicida* inferred by *vapA* genotyping

**DOI:** 10.1093/femsle/fnz074

**Published:** 2019-04-12

**Authors:** Snorre Gulla, Sion Bayliss, Bryndís Björnsdóttir, Inger Dalsgaard, Olga Haenen, Eva Jansson, Una McCarthy, Felix Scholz, Maaike Vercauteren, David Verner-Jeffreys, Tim Welch, Tom Wiklund, Duncan J Colquhoun

**Affiliations:** 1 Fish Health Research Group, Norwegian Veterinary Institute, Oslo, Norway; 2 The Milner Centre for Evolution, Department of Biology & Biotechnology, University of Bath, Bath, England; 3 Matis, Reykjavik, Iceland; 4 National Institute of Aquatic Resources, Technical University of Denmark, Lyngby, Denmark; 5 NRL for Fish Diseases, Wageningen Bioveterinary Research, Lelystad, the Netherlands; 6 Department of Animal Health and Antimicrobial strategies, National Veterinary Institute (SVA), Uppsala, Sweden; 7 Marine Scotland Science, Marine Laboratory, Aberdeen, Scotland; 8 FishVet Group Ireland, Galway, Ireland; 9 Department of Pathology, Bacteriology and Avian Diseases, Faculty of Veterinary Medicine, Ghent University, Merelbeke, Belgium; 10 Cefas, Weymouth Laboratory, Weymouth, England; 11 National Center for Cool and Cold Water Aquaculture, Agricultural Research Service, US Department of Agriculture, Kearneysville, West Virginia, USA; 12 Laboratory of Aquatic Pathobiology, Environmental and Marine Biology, Åbo Akademi University, Turku, Finland; 13 Department of Biological Sciences, University of Bergen, Bergen, Norway

**Keywords:** aquaculture, bacterial fish pathogen, *Aeromonas salmonicida*, vapA*/*A-layer, genotyping, host specificity

## Abstract

A recently described typing system based on sequence variation in the virulence array protein (*vapA*) gene, encoding the A-layer surface protein array, allows unambiguous subtyping of *Aeromonas salmonicida*. In the present study, we compile A-layer typing results from a total of 675 *A. salmonicida* isolates, recovered over a 59-year period from 50 different fish species in 26 countries. Nine novel A-layer types (15–23) are identified, several of which display a strong predilection towards certain fish hosts, including e.g. Cyprinidae and Pleuronectidae species. Moreover, we find indications that anthropogenic transport of live fish may have aided the near global dissemination of two cyprinid-associated A-layer types. Comparison of whole genome phylogeny and A-layer typing for a subset of strains further resulted in compatible tree topologies, indicating the utility of *vapA* as a phylogenetic as well as an epizootiological marker in *A. salmonicida*. A Microreact project (microreact.org/project/r1pcOAx9m) has been created, allowing public access to the *vapA* analyses and relevant metadata. In sum, the results generated provide valuable insights into the global population structure of *A. salmonicida*, particularly in relation to its piscine host spectrum and the geographic distribution of these hosts.

## Teaser

Sub-lineages of the fish-pathogenic bacterium *Aeromonas salmonicida* display specific host preferences.

## INTRODUCTION

*Aeromonas salmonicida* infections have caused significant problems and economic losses in commercial farming of a large number of cultivated fish species (Austin and Austin 2012). To date, *A. salmonicida* represents one of the most intensively studied fish pathogenic bacteria. Historically, most of the attention has fallen on *A. salmonicida* subsp. *salmonicida* (Lehmann and Neumann 1896; Griffin, Snieszko and Friddle 1953), commonly referred to as ‘typical’ *A. salmonicida*, which primarily causes disease in salmonids. In recent years however, the highly diverse group of non-subsp. *salmonicida* strains, commonly known as ‘atypical’ and mainly isolated from non-salmonid fish, has come under increasing scrutiny. The collective ‘atypical’ group includes, but is not limited to, the four other validly described subspecies, i.e. *achromogenes*, *masoucida*, *smithia* and *pectinolytica* (Martin-Carnahan and Joseph 2005).

For many years, professionals were unable to systematise the phenotypically diverse range of atypical *A. salmonicida* isolates (Austin *et al*. 1998; Wiklund and Dalsgaard 1998). Recently however, a simple typing scheme was introduced (Gulla *et al*. 2016), based on sequence variation in a hypervariable region of the virulence array protein (*vapA*) gene (henceforth termed ‘partial *vapA*’). In *A. salmonicida*, this gene encodes the paracrystalline surface protein commonly referred to as the A-layer (Udey and Fryer 1978; Kay *et al*. 1981; Evenberg *et al*. 1982; Chu *et al*. 1991), the auto-agglutinating properties of which is responsible for the ‘friable’ colony morphology commonly observed following cultivation on solid media. Based on partial *vapA* sequences, 333 *A. salmonicida* isolates of varying origin could be differentiated into 14 discrete clusters (‘A-layer types’) and five singletons (Gulla *et al*. 2016).

While a number of the previously identified A-layer types displayed a strong association with certain species of fish, the number of different fish hosts and geographic locations investigated in that study were limited. The aim of the present study was, therefore, to comprehensively assess the biogeography of fish-pathogenic *A. salmonicida* and further investigate the putative link between A-layer type and fish host.

## MATERIALS AND METHODS

Metadata and *vapA* (or genome) accession numbers on all *A. salmonicida* isolates included in this study are provided in Table S1 (Supporting Information). The present study raises the total number of publically available *A. salmonicida* partial *vapA* sequences to 675. The studied isolates were recovered between 1959 and 2017 from 26 countries (five continents) and at least 50 fish species (24 families).

Lyophilised or cryopreserved stock cultures were revived by seeding onto appropriate culturing media (e.g. 5% bovine blood agar) followed by incubation at 22°C for 2–4 days prior to further processing. While all isolates had previously been identified as *A. salmonicida* in the respective laboratories of recovery, the authenticity of these identities were not, as part of the present study, verified through a unified array of phenotypic assessments. However, successful PCR amplification of the *vapA* gene and a clustering alongside confirmed *A. salmonicida* strains in the resulting partial *vapA* tree (see below) was in itself considered confirmatory evidence for their species affiliation.

DNA extraction, PCR and Sanger sequencing was conducted as previously described (Gulla *et al*. 2016), with the exception of sequences obtained directly from the NCBI GenBank or extracted from genome assemblies (Gulla *et al*., unpublished). Briefly, PCR and sequencing employed primers *vapA* F2 and R3, which flank the hypervariable *vapA* gene region corresponding to nt 1497304–1497708 in the circularised genome of strain A449 (assembly accession no. GCA_000196395.1). Sequence alignments were conducted in ClustalX v2.1 (Larkin *et al*. 2007). Maximum Likelihood (ML) trees were constructed using PhyML v3.0 (Guindon *et al*. 2010), employing the Smart Model Selection option (Lefort, Longueville and Gascuel 2017), and the Approximate Likelihood-Ratio test (Anisimova and Gascuel 2006) for branch support estimation. ML trees were edited in FigTree v1.4.3 (tree.bio.ed.ac.uk/software/figtree) and/or MEGA X (Kumar *et al*. 2018) prior to downstream applications. Isolates displaying frameshifting *vapA* indels (Belland and Trust 1987; Gustafson, Chu and Trust 1994) were, for practical reasons, excluded from the material. Isolates were classified according to the system published by Gulla *et al*. (2016), with previously undescribed clusters being successively awarded new type designations.

A partial *vapA* ML tree file, together with metadata for all examined isolates, was uploaded to the Microreact (Argimón *et al*. 2016) web application that can be publically accessed through a unique project link at microreact.org/project/r1pcOAx9m. The geographic origins of isolates were defined by prioritising the most accurate information available (e.g. estuary > river > province > country). In the particular case of Norwegian isolates, aquaculture sites were anonymised by using coordinates representing the ‘centre’ of the relevant municipality or county.

A tree file comparing 29 *A. salmonicida* genome assemblies available from NCBI GenBank was exported from the NCBI Tree Viewer application, and an ML tree based on partial *vapA* sequences from the same strains was created for comparison. Subsp. *pectinolytica* strains, and other representatives lacking the *vapA* gene (Lund, Espelid and Mikkelsen 2003; Merino *et al*. 2015; Gulla *et al*. 2016), were excluded.

## RESULTS AND DISCUSSION

An A-layer typing scheme for the fish pathogen *A. salmonicida*, based on sequence heterogeneity in the *vapA* virulence gene, has recently been demonstrated as a cost-effective, rapid and unambiguous tool for genetic subtyping of this bacterium (Gulla *et al*. 2016). The method has since been employed by several investigators for characterisation of *A. salmonicida* strains (e.g. Long *et al*. 2016; Du *et al*. 2017; Scholz *et al*. 2017; Vercauteren *et al*. 2017). In the present study, we compared *vapA* sequences from an extended *A. salmonicida* collection (675 isolates) of worldwide origin, recovered over six decades from a broad range of fish hosts. Nine novel A-layer types were identified and the known geographical range of previously described A-layer types was expanded. Several A-layer types found over large geographic areas remain exclusively associated with only single or a limited number of fish host lineages. The geographic distribution of individual types is likely dependent on the availability of susceptible hosts, and in some cases we found that anthropogenic activity has presumably played a significant role for spatial dissemination.

ML tree analysis performed on *A. salmonicida* partial *vapA* sequences identified eight singletons and 23 discrete clusters, each comprising two or more isolates (Table 1 and Figure S1, Supporting Information). The nine novel clusters identified represent A-layer types 15 through 23. A *vapA* homolog identified by BLAST within two recently published Aeromonad genomes (genome assembly accession no.: GCA_0 017 29085.1 (S-layer: OEC65338) and GCA_0 017 29005.1 (S-layer: OEC54980)) (Vázquez-Rosas-Landa *et al*. 2017), displaying 65–74% pairwise identity with *vapA* sequences from *A. salmonicida*, provided an ideal non-*A. salmonicida* outgroup, which has previously been lacking.

**Table 1.** Observed characteristics of designated A-layer types. See Table S1 and/or Microreact project (microreact.org/project/r1pcOAx9m) for extended metadata on all isolates.

A-layer type	No. of isolates	Main hosts (families) involved (%)^a^	Known geographic distribution^b^	Temporal span	Associated subspecies	Representative strain^c^
1	97	Salmonidae (68)	Atlantic (NW, NE), Pacific (NW, NE)	1963–2016^d^	*salmonicida*	ATCC33658
2	79	Pleuronectidae (86)	Norway	1987–2016		NVI-04953
3	93	Salmonidae (45), Gadidae (41)	Atlantic (NW, NE)	1962–2016^d^	*achromogenes*	NCIMB1110
4	23	Anarhichadidae (48), Zoarcidae (17)	Atlantic (NW, NE)	1981–2014^d^		CECT5200
5	52	Labridae (94)	Europe	2008–2017		NVI-08017
6	164	Labridae (55), Cyclopteridae (38)	Europe	1987–2017		NVI-08013
7	20	Salmonidae (55), Sebastidae (20)	Pacific (NW, NE, SE)	1969–2016^d^	*masoucida*	NBRC13784
8	7	Salmonidae (86)	Norway	2002–2016		NVI-06457
9	17	Salmonidae (82), Cyprinidae (12)	Europe	1976–2014		NVI-04214
10	13	Cyprinidae (92)	Europe, USA, Australia	1979–2006		NVI-03454
11	7	Salmonidae (86)	Northern Europe	1985–2013		NVI-06449
12	10	Salmonidae (90)	Europe	1987–2008	(*smithia*)^e^	JF4097
13	3	Salmonidae (100)	Eastern Canada	1987^d^		NVI-03080
14	4	Salmonidae (100)	Norway	1990–2014		NVI-01843
15	24	Pleuronectidae (100)	Europe	1992–2016		2CE
16	8	Esocidae (62), Salmonidae (38)	Northern Europe	1984–2012		5G13–9
17	7	Scophthalmidae (100)	Europe	1990–1994		NVI-01844
18	17	Pleuronectidae (100)	Europe	1989–2009		2F15–17
19	11	Cyprinidae (100)	Europe, USA	1994–2015		12002514–3
20	4	Order: Anguilliformes (75)	Denmark, South-Korea	1992–2006		AS03
21	3	Salmonidae (100)	Chile	1999^d^		NVI-03995
22	2	Cyprinidae (100)	Europe	1981–1997^d^		NVI-03062
23^f^	2	Pleuronectidae (100)	Denmark	1992–1996		14

a Excluding isolates of unknown origin.

b Only considering isolates involved in the present study. Abbreviations: northwest (NW), northeast (NE), southeast (SE).

c Reference cultures where available.

d Subject to some uncertainty.

e Type-strain located marginally outside A-layer type 12.

f Mismatch in 3′-end of R3 primer; partial *vapA* extracted from genome assemblies (Gulla *et al*., unpublished).

Unsurprisingly, the previous grouping threshold of ≥98% partial *vapA* pairwise sequence identity for A-layer type inclusion (Gulla *et al*. 2016) could not be consistently enforced, an inevitable consequence of the increasing spectrum of *A. salmonicida* strains investigated. Definition of a universal identity threshold for A-layer type cluster partitioning therefore became impossible, but all isolates could nevertheless be readily assigned to single A-layer types based on their relative positioning within the tree.

**Figure 1.**
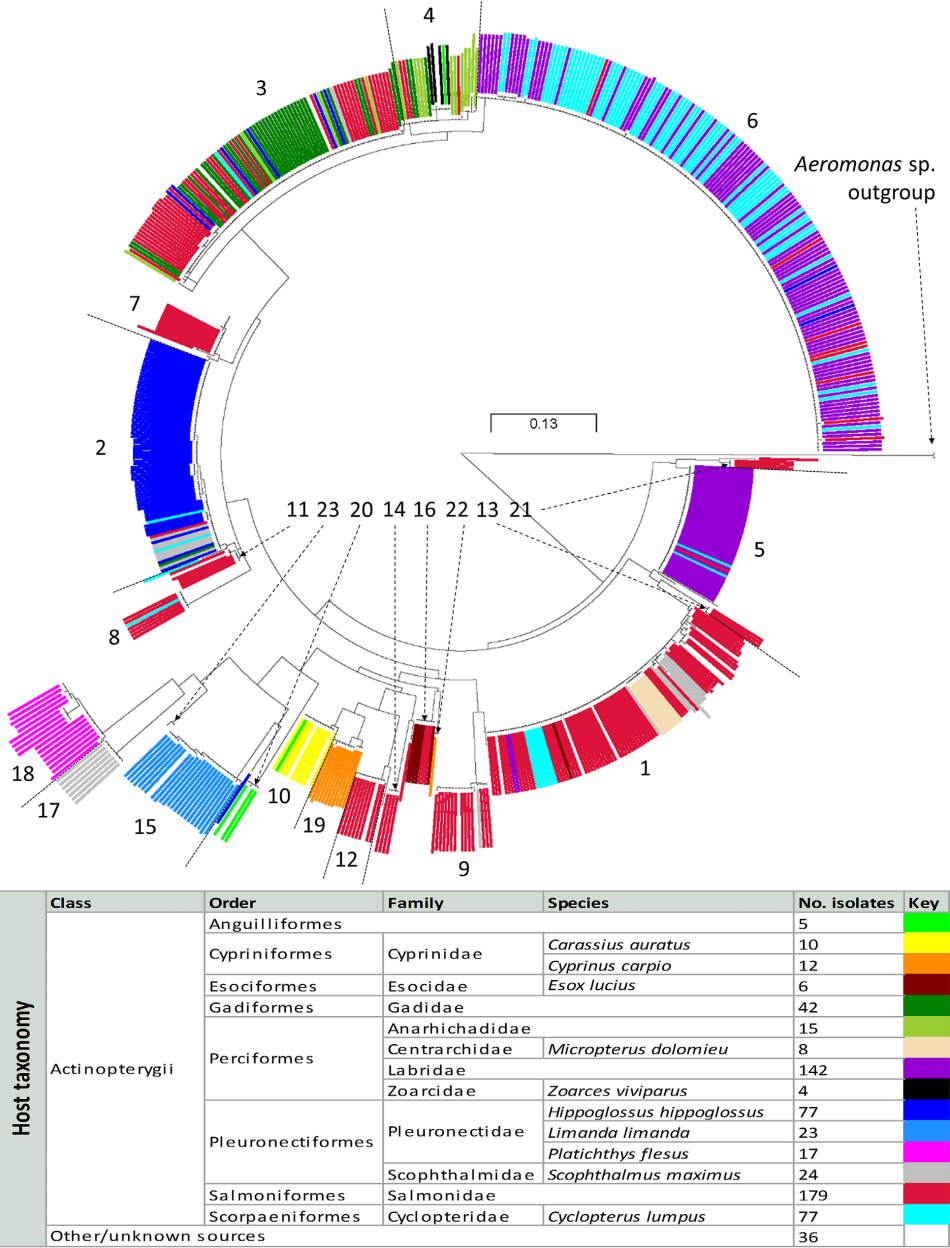
*Aeromonas salmonicida* A-layer type clustering in relation to host fish species. The circular ML tree is based on partial *vapA* sequences from 675 *A. salmonicida* isolates and two *Aeromonas* sp. outgroup strains. The tree visualises how isolates recovered from selected taxonomic fish groups (indicated by colour; see legend) in most of the cases belong to only a limited number of A-layer type clusters (numbered in the tree). Tree exported from microreact.org/project/r1pcOAx9m. For rectangular high resolution tree with strain identifiers and branch support, see Figure S1.

Most A-layer types could be clearly linked to a particular host (i.e. taxonomic fish lineage; Table 1), and vice versa, with most of the examined fish hosts represented in only one or a few *vapA* clusters (Fig. 1). For instance, all isolates recovered from the fish species common dab (*Limanda limanda*), European flounder (*Platichthys flesus*) and goldfish (*Carassius auratus*)—in each case involving ≥10 isolates, recovered from ≥3 countries over a period of ≥20 years—clustered exclusively in separate, single A-layer types.

**Figure 2.**
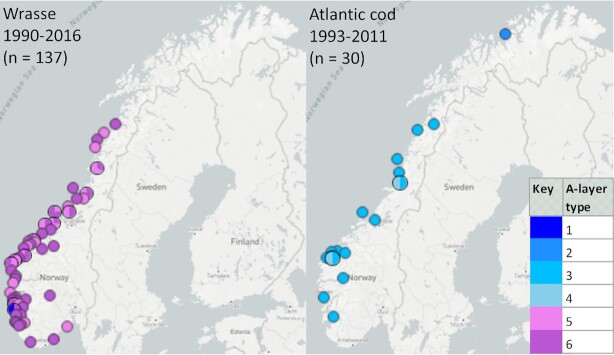
*Aeromonas salmonicida* from wrasse and Atlantic cod in Norway. The spatiotemporal origins and A-layer types (see legend) of investigated isolates are shown to the left and right, respectively. Coinciding sampling points over the same time period indicate that the host-associated representation of A-layer types presumably has a biological basis. Maps exported from microreact.org/project/r1pcOAx9m.

Further, A-layer types recovered from marine fish along the Norwegian coast, such as wrasse (Labridae) and Atlantic cod (*Gadus morhua*) (Fig. 2), were heavily biased towards these particular host species despite coinciding/overlapping spatiotemporal origins. Taken together, these findings strongly suggest that the observed host/A-layer type relationships have a biological basis and are not founded upon temporal and/or geographic sampling biases.

In broader geographic terms, some A-layer types appear restricted by the spatial ranges of their natural, wild-living hosts, while others show signs of dissemination linked to anthropogenic activity. The former situation is exemplified by types 15, 17 and 18, which have all been found exclusively in coastal Northern Europe, from common dab, turbot (*Scophthalmus maximus*) and European flounder, respectively. In contrast, transport of live fish has presumably contributed towards the near global distribution of types 10 and 19, respectively, associated with the domesticated and extensively traded freshwater fish species koi/common carp (*Cyprinus carpio*) and goldfish. In other cases, such as for type 1 from (predominantly) cultivated Salmonidae species globally, and type 7 from various fish species across the Pacific Ocean, the historic epizootiological events underlying their geographic spread is less clear. Nevertheless, these findings may serve as a reminder regarding the possible biosecurity risks arising in relation to transport of live animals.

**Figure 3.**
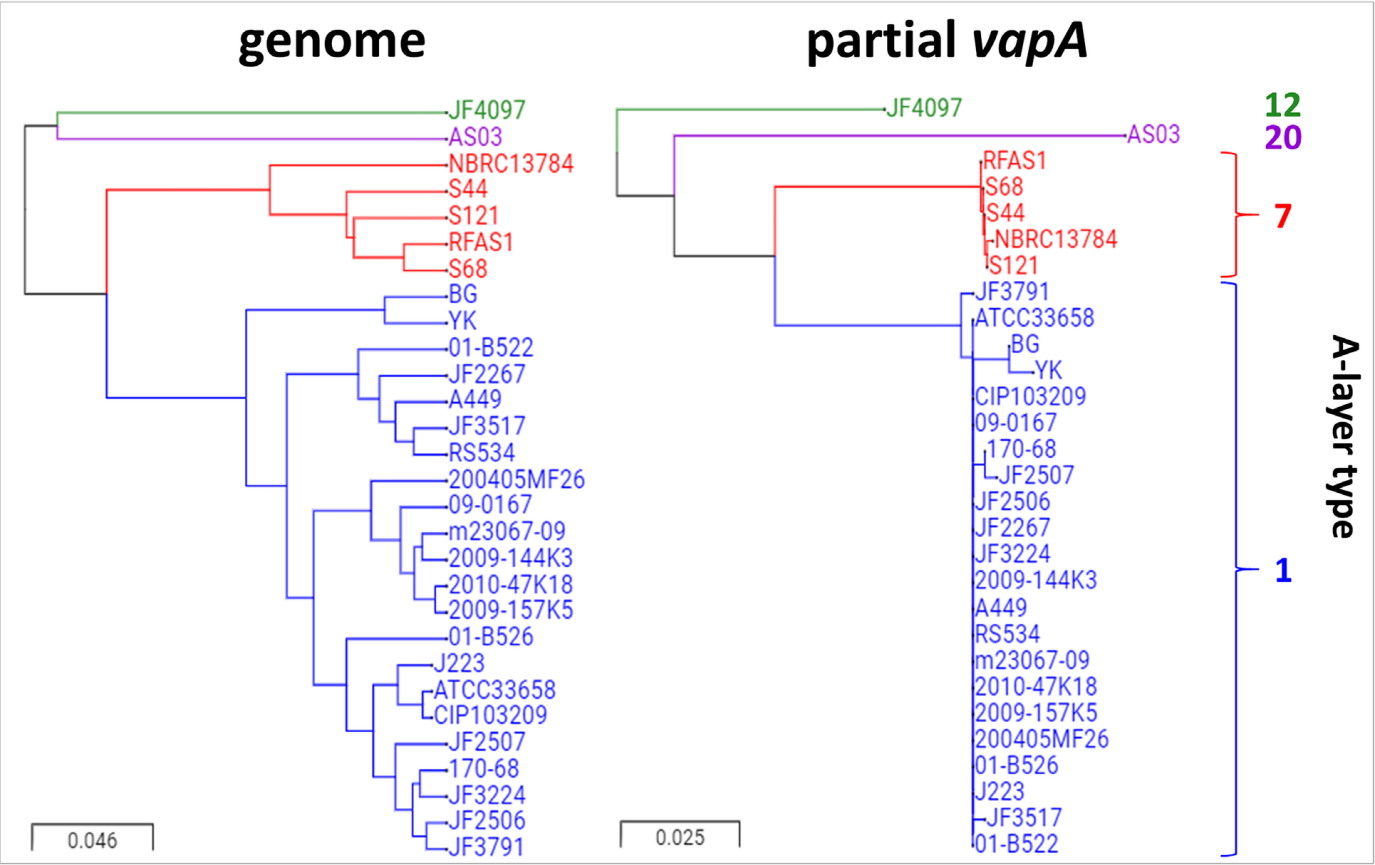
Comparison of *Aeromonas salmonicida* whole genome phylogeny and A-layer type clustering. Twenty-nine strains are compared, with branches and labels coloured according to their affiliated A-layer type (see far right). The consistent clustering indicates the potential of *vapA* as a representative phylogenetic marker in *A. salmonicida*.

Comparison of whole genome phylogeny and partial *vapA* genotype for a subset of strains revealed consistent clustering (Fig. 3). This indicates limited recombination in the *vapA* gene between distantly related lineages, and suggests the potential of *vapA* as a suitable phylogenetic marker in *A. salmonicida*. It should be noted, however, that the analysed whole genome dataset was strongly biased towards A-layer types 1 and 7 (subsp. *salmonicida* and *masoucida*, respectively), and broader conclusions should therefore be reserved pending analysis of a more comprehensive genome dataset.

Notably, recent years have brought several reports describing recovery of ‘mesophilic’ *A. salmonicida* from various sources other than diseased fish. These include, amongst others, subsp. *pectinolytica* specimens from polluted freshwater (Pavan *et al*. 2000) and also a few clinical isolates from human patients (Ruppé *et al*. 2018; Vincent *et al*. 2019). The current taxonomic subdivision of *A. salmonicida*, into a single mesophilic and four psychrophilic validly described subspecies, is questioned by core genome phylogenies, which reveals a marked genomic separation between the two phenotypes, with the psychrophilic lineage being by far the most genetically conserved (Vincent *et al*. 2017, 2019). As the *vapA* gene was apparently acquired by the psychrophilic lineage subsequent to the bifurcation of the two, A-layer typing remains limited to investigation of primarily fish-associated *A. salmonicida* (Gulla *et al*. 2016).

In summary, the present study substantially expands the number of *A. salmonicida* isolates (in terms of both host species and geographic origin) evaluated by A-layer typing. This has provided further support for the existence of discrete genetic subtypes of *A. salmonicida* displaying distinct, often highly specific, fish host affinities. The observed geographic distribution of some such subtypes presumably reflects anthropogenic activities having involved transport of live fish. We also find indications that the partial *vapA* gene may represent a suitable phylogenetic marker for deeper underlying population genetics amongst psychrophilic *A. salmonicida*. Further studies involving whole genome sequence analysis of a substantially extended number of *A. salmonicida* strains from diverse fish species and disparate geographic origins, covering the spectrum of novel A-layer types described here, are now required to investigate the situation.

To allow general access to data generated under the current project, the *vapA* tree and relevant metadata for the analysed *A. salmonicida* dataset was uploaded into Microreact (Argimón *et al*. 2016), and can be accessed through the project link microreact.org/project/r1pcOAx9m. This web application provides a user friendly platform for sharing, visualising and interactively exploring genetic epidemiological data (consult Argimón *et al*. 2016 for detailed features).

## Supplementary Material

Supplemental Files
