# Supplementary figures and images for: Biogeography of the fish pathogen Aeromonas salmonicida inferred by vapA genotyping
Source: FEMS Microbiol Lett. 2019 Apr 12;366(7):fnz074. doi: 10.1093/femsle/fnz074 (PMC6502549; doi:10.1093/femsle/fnz074)

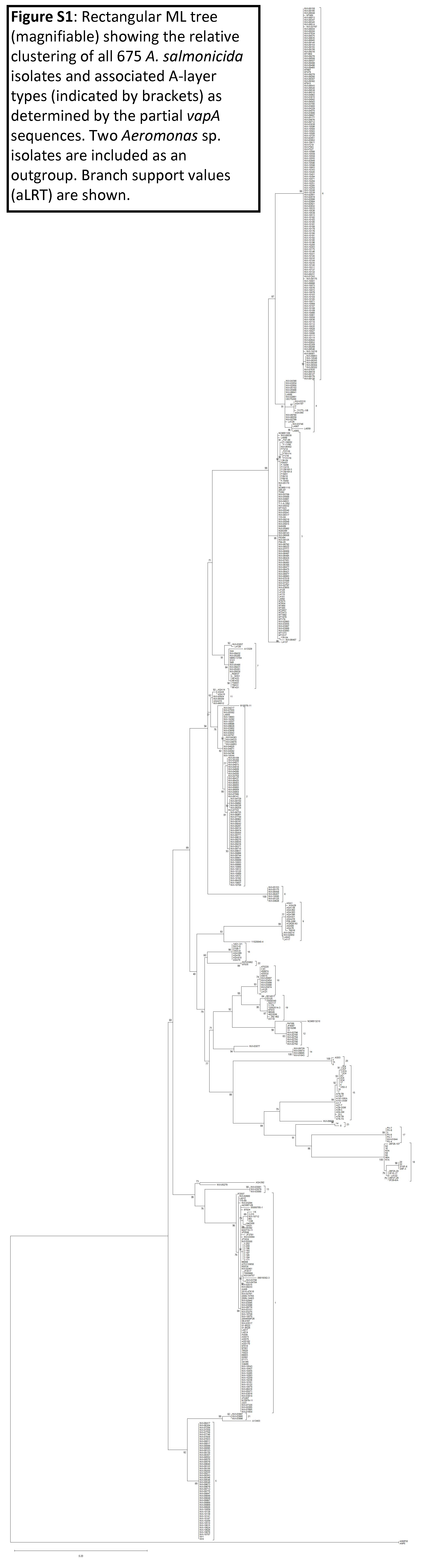

Supplement: Supplemental Files [file fnz074_supplemental_files.zip › FigS1.tif]
